# Mini-review: *In vitro* Metabolic Engineering for Biomanufacturing of High-value Products

**DOI:** 10.1016/j.csbj.2017.01.006

**Published:** 2017-01-19

**Authors:** Weihua Guo, Jiayuan Sheng, Xueyang Feng

**Affiliations:** Department of Biological Systems Engineering, Virginia Polytechnic Institute and State University, Blacksburg, VA 24061, United States

**Keywords:** Cell-free, Biosynthesis, Metabolic pathways, Design-build-test cycle

## Abstract

With the breakthroughs in biomolecular engineering and synthetic biology, many valuable biologically active compound and commodity chemicals have been successfully manufactured using cell-based approaches in the past decade. However, because of the high complexity of cell metabolism, the identification and optimization of rate-limiting metabolic pathways for improving the product yield is often difficult, which represents a significant and unavoidable barrier of traditional *in vivo* metabolic engineering. Recently, some *in vitro* engineering approaches were proposed as alternative strategies to solve this problem. In brief, by reconstituting a biosynthetic pathway in a cell-free environment with the supplement of cofactors and substrates, the performance of each biosynthetic pathway could be evaluated and optimized systematically. Several value-added products, including chemicals, nutraceuticals, and drug precursors, have been biosynthesized as proof-of-concept demonstrations of *in vitro* metabolic engineering. This mini-review summarizes the recent progresses on the emerging topic of *in vitro* metabolic engineering and comments on the potential application of cell-free technology to speed up the “design-build-test” cycles of biomanufacturing.

## 1 Introduction

For decades, scientists and engineers use metabolic engineering as a powerful approach to optimize industrial fermentation processes through the introduction of directed genetic changes using recombinant DNA technology. This has become an attractive, sustainable way to produce molecules [1], [2], [3], especially when chemical synthesis is difficult [4], [5]. Metabolic engineering aims to endow cells with improved properties and performance [6] while synthetic biology could create new biological parts, modules, devices and systems, in addition to re-engineering cellular components and machinery that nature has provided [7]. Through the integration of metabolic engineering and synthetic biology, efficient microbial cell factories can be constructed to produce biofuels, biomaterials and drug precursors [8].

As high-valued products, biologically active compound is one kind of the most attractive engineering targets nowadays because many of them demonstrate important pharmacological activities or biotechnological significance [9]. However, due to the complexity of their structures which contains multiple chiral centers and labile connectivity [10], researchers seek microbial production instead of total chemical synthesis or semisynthesis from isolated precursors. However, these products often lack optimal production titer and high yield. Till now, except for a few examples such as introducing heterologous pathways into yeast for the large scale production of an anti-malaria drug artemisinin [11], few valuable biologically active compounds could be produced at high yield and reach into the stage of large-scale biomanufacturing. Commodity chemicals is another large group of chemicals that attracts researchers to use cell-based metabolic engineering for manufacturing, mainly due to concerns of depleting fossil fuels and climate changes [12]. Biomass produced from plants is the most abundant renewable resource and is considered to be the cost-competitive energy and carbon sources that could be converted to produce biofuels and biochemicals instead of fossil fuels [13]. Recent breakthroughs in synthetic biology and metabolic engineering led to the production of a series of bulk chemicals such as 1,4-butanediol [14] and isobutanol [15]. However, cell proliferation is the primary goal of microorganisms while bioconversions are the side effects. These inherent constraints of living microorganisms prevent them from implementing some important chemical reactions (*e.g.*, H_2_ production from glucose and water) and prohibit them from achieving the theoretical yield of commodity chemicals.

The unsatisfactory results of large-scale biomanufacturing of high-value products and commodity chemicals are largely due to two challenges: complex cell-wide regulation of metabolic pathways, and difficulty in balancing biosynthesis of target products and innate cell physiology. First, a lot of organisms are difficult to be engineered because of unknown regulation patterns and the lack of engineering tools for non-model organisms [16]. Even for model microorganisms like *Escherichia coli* and *Saccharomyces cerevisiae*, which are well studied and equipped with a broad spectrum of biomolecular tools to allow metabolic engineering easily, the effects of heterologous expression of pathways are often unpredictable to guarantee a high productivity, as witnessed in metabolic engineering of *S. cerevisiae* to produce n-butanol [17] and engineering carbon dioxide fixation in *E. coli* [18]. In order to identify optimal biosynthetic systems and discover the best sets of enzymes, the “design-build-test (DBT)” cycles [19] are often used. However, the DBT cycles usually take months to finish, as culturing cells is time consuming. Second, a key challenge in metabolic engineering is balancing the tug-of-war that exists between the cell's physiological and evolutionary objectives on one side and the engineer's process objectives on the other [20]. Such conflict of resource allocation sometimes cannot be well addressed and toxic intermediates could be built up in the unbalanced pathway thus the manufacturing of high-value products often ends up with a low titer and yield and a high cost.

**Fig. 1:**
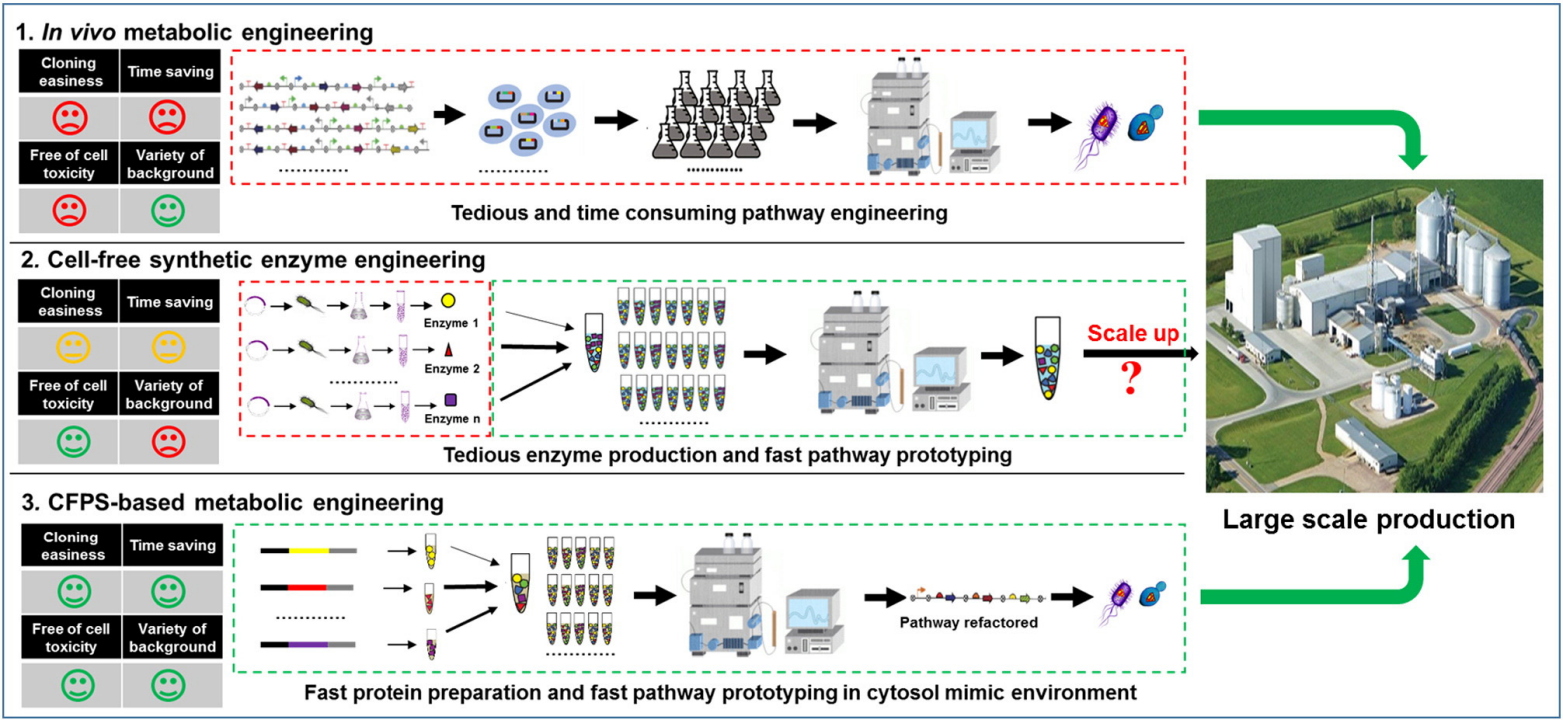
Summary of *in vitro* metabolic engineering (ME) approaches. 1. *In vivo* metabolic engineering, in which model microorganisms like *Escherichia coli* and *Saccharomyces cerevisiae* are often accompanied with inefficient and time-consuming pathways construction, transformation and fermentation; 2. Cell-free synthetic enzyme engineering, which allows fast pathway prototyping; however, molecular cloning and enzyme production could be time consuming and the high cost associated with production could make the process scale-up questionable. 3. The cell-free protein synthesis (CFPS)-based metabolic engineering, which could accelerate the pathway prototyping in a cytosol mimic environment by using enzymes that are directly produced in a cell-free system and assembling pathways in a “mix-and-match” fashion.

Many emerging technologies seek to address these challenges. Among them, cell-free biotechnology is one of the promising approaches that offer complementary advantages to *in vivo* metabolic engineering, especially in its potentials of speeding up the DBT cycles [21]. In general, the cell-free biotechnology bypasses the cell growth, and thus becomes time saving to permit more DBT cycles and avoids the conflict of resource allocation between cell growth and biosynthesis of target products. The cell-free biotechnology also uses an open reaction environment, which allows the easy and precise adjustment of components such as cofactors and intermediates during a biosynthetic reaction [22]. The cell-free biotechnology was first developed in 1961 for the purpose of elucidating the codon usage [23] and was repurposed for protein production since the end of the 1990s [24], [25], [26], [27]. Recently in late 2000s, the cell-free biotechnology was further re-engineered to produce both biologically active compound and commodity chemicals [28], [29], [30]. In this mini-review, we summarized the experimental set-up and computational modeling of two *in vitro* metabolic engineering approaches: cell-free synthetic enzyme engineering and cell-free protein synthesis (CFPS)-based metabolic engineering (Fig. 1).

## 2 Cell-free Synthetic Enzyme Engineering

The principle of cell-free synthetic enzyme engineering is to purify the individual enzymes of a biosynthetic pathway, reconstitute the pathway and study its performance *in vitro*. For more than 100 years, biologists have sought to excise complete enzymatic pathways from their native cellular environments for biochemistry research [31]. *In vitro* analysis of metabolic pathways is becoming a powerful method to gain fundamental understanding of biochemical transformations, to reveal the mechanisms of enzymatic reactions and kinetics, and to identify key metabolites and feedback control of enzyme activities.

### 2.1 Functional Investigation of Natural Enzymes and Metabolisms

As a powerful method to investigate natural enzymes and metabolisms, some remarkable achievements have been reported. One remarkable example is the study of the bacterial fatty acid synthases. Although being investigated extensively at the genetic and enzymatic level, it is still not easy to manipulate enhanced production of specific fatty acids because of the complex cell-wide regulation of fatty acid synthesis. In 2010, Liu et al. revealed the strong dependence of fatty acid synthesis on malonyl-CoA availability and several important phenomena in fatty acid synthesis by a quantitative investigation of the fatty acid biosynthesis and regulation in a cell-free synthetic enzyme system [32]. Following these discoveries, Yu and colleagues reported an *in vitro* reconstitution of the fatty acid synthase derived from *E. coli* by overexpressing all nine fatty acid biosynthesis (Fab) enzymes and the acyl carrier protein (ACP) in the natural *E. coli* host, and purifying the enzymes to homogeneity. Upon supplementing the ten protein species with acetyl-CoA, malonyl-CoA and NADPH, C14-C18 fatty acids were observed in the system, evidenced by ^14^C-isotope incorporation experiments and subsequently *via* UV-spectrophotometry [33]. The reconstituted multi-enzyme system has also highlighted that the fine-tuning of each individual components could substantially influence the partitioning between unsaturated and saturated fatty acid products. Similar to fatty acid biosynthesis, another pathway which synthesizes isoprenoids as key metabolites in both primary and secondary metabolisms, was reconstituted *in vitro*. Basically, in order to develop a route to synthesize the jet fuel farnesene, Zhu and colleagues reconstituted the mevalonate (MVA) pathway in a cell-free synthetic enzyme system *in vitro* by expressing and purifying eight enzymes of the MVA pathway as well as the α-farnesene synthase from an *E. coli* host [34]. The purified enzymes worked in tandem with the requisite NADPH and ATP cofactors to produce farnesene, as confirmed by gas chromatography–mass spectrometry. It was found that the isopentenyldiphosphate (IPP) isomerase was the most influential factor on the turnover rate of this pathway.

In addition to bacterial pathways, some eukaryotic pathways were also reconstituted *in vitro*. The biosynthetic pathways of dhurrin, which plays an important role in plant defense against pathogens [35], and camalexin, which is cytotoxic against aggressive prostate cancer cell lines [36], have been studied in cell-free synthetic enzyme system. Kahn and colleagues reconstituted the entire dhurrin biosynthetic pathway *in vitro* using enzymes from the natural host organism [37]. Through tedious enzyme purification processes, the researchers were able to obtain all three enzymes, CYP79, glycosyltransferase and P450ox, in the microsomal fraction of the *Sorghum bicolor* lysates. It was found that the microsomal environment could allow functional expression of catalytically active CYP79 and P450ox, and thus dhurrin synthesis was observed by radioactive TLC analysis when combining the three enzymes with ^14^C-tyrosine, UDP-glucose, and NADPH. In another study, camalexin pathway was constructed *in vitro* by purifying three enzymes: CYP79B2, which catalyzes decarboxylation and N-hydroxylation of tryptophan to indole-3-acetaldoxamine (IAOx); a second P450 enzyme, which was previously unknown and is believed to catalyze an oxidative coupling of cysteine to IAOx; and CYP71A15, which decarboxylates and cyclizes the resulting cysteine-indole-3-acetonitrile (Cys-IAN) compound to form the thiazole ring structure within camalexin. By using a combination of gene expression data and protein sequence analysis, Klein and coworkers were able to identify a P450 enzyme capable of performing the C–S coupling reaction and to reconstitute the entire camalexin pathway *in vitro* for the first time [38].

### 2.2 Production of Biocommodities

Perhaps a more advanced and systematic application of cell-free synthetic enzyme engineering, especially for reconstituting long biosynthetic pathways that involves a large number of enzymes for chemical production purposes [12], is the development of Synthetic Pathway Biotransformation (SyPaB) [8]. The development cycle of SyPaB is composed of five parts: (i) pathway reconstruction, (ii) enzyme selection, (iii) enzyme engineering, (iv) enzyme production, and (v) process engineering. The entire SyPaB process can be improved in an iterative manner, which allows gradual improvement to an efficient industrial process. The DBT cycles of SyPaB have proven to be much faster than the *in vivo* systems [8]. As demonstrated in the pioneer work of high-yield cell-free hydrogen production in Zhang's lab, bulk chemicals could be potentially manufactured in a cost-effective manner [39]. This cell-free hydrogen synthetic pathway contains four modules: 1) a chain-shortening phosphorylation reaction for producing glucose-1-phosphate (G-1-P) catalyzed by glucan phosphorylase; 2) conversion of G-1-P to glucose-6-phosphate (G-6-P) catalyzed by phosphoglucomutase; 3) a pentose phosphate pathway containing 10 enzymes for producing 12 NADPH per G-6-P; and 4) hydrogen generation from NADPH catalyzed by hydrogenase. The maximum hydrogen production rate reached 3.92 mmol of hydrogen per hour per liter of reactor. When cellobiose was used as the substrate with a reaction time of 150 h for a complete reaction, the overall yield of H_2_ was 11.2 mol per mole of anhydroglucose unit of cellobiose, corresponding to 93.1% of the theoretical yields. This yield was more than 2 times higher than the yield from microbial fermentations which is limited to 4 H_2_ per mole of glucose [40], [41]. In another study, Honda and his coworkers designed an *in vitro* non-natural, ATP balanced pathway for n-butanol production from glucose [42]. This pathway comprised 16 thermostable enzymes with three modules: 1) generation of two pyruvate and two NADH from one glucose molecule without ATP accumulation, 2) generation of acetyl-CoA from pyruvate; and 3) *n*-butanol production from two acetyl-CoAs. As a result, one molecule of glucose was able to produce one molecule of n-butanol, two molecules of CO_2_ and one molecule of water. Recently, Opgenorth et al. described a robust, efficient synthetic glucose breakdown pathway and implemented it to produce bioplastic PHB [43]. The designed PBG cycle produces a net of 2 acetyl-CoA, 4 NAD(P)H, and 0 ATP for each glucose molecule and 66.6% theoretical molar yield of carbon due to the release of CO_2_. Because the PBG pathway generated more reducing equivalents than are needed to produce PHB (4 NADPH per glucose produced but only 1 NADPH needed), the authors designed a NAD(P)H purge valve regulatory nodes which composed of a mixture of dehydrogenases to prevent the buildup of NADPH. Reactions were initiated with 60.7 glucose and continuously monitored in 10-h cycles by absorbance at 600 nm. It was observed that by the end of the third cycle, the reaction stopped by the depletion of glucose with a production of 57 ± 6 mM PHB (monomer equivalents), corresponding to a 94% yield. When reactions were initiated with 109.2 mM glucose, the system maintained > 50% of the maximum activity over the entire 55 h run at room temperature and generated 93.8 ± 6.1 mM PHB, corresponding to an 86% yield. The high yield emphasized the importance of cofactor recycling for SyPaB system. Compared to the microbial production of PHB using *Cupriavidus necator* [44], cell-free synthetic enzyme engineering has higher (94%) yield but lower titer (~ 10 g/L) than microbial bioprocess (60% yield and 83 g/L titer).

In order to further understand and predict the performance of biological systems, computational modeling has been commonly applied [45]. Cell-free synthetic enzyme engineering can be modeled at multiple levels from molecules to modules to systems [46], [47]. Compared to *in vivo* cell metabolism, the relative simplicity of *in vitro* biological systems makes them far easier to simulate processes and predict optimal enzyme ratios for maximizing product yield and accelerating volumetric productivity. This simplicity could be concluded into five aspects: 1) it is free of complex transcriptional or translational regulations; 2) lower background noises in the defined system; 3) accurate measurements of metabolic components; 4) better defined model parameters, and 5) smaller modeling scales compared to *in vivo* systems. With the development of high-speed computers and the accumulation of huge biological data, numerous computational tools have been developed to simulate the *in vivo* cell metabolism and to facilitate the design of *in vivo* metabolic engineering [48]. One of the most famous computational modeling approaches is the flux balance analysis, which simulates cell metabolism at genome-scale to provide the potential target genes for better production of chemicals [49], [50]. In addition, another commonly used computational model is the kinetic model, in which a group of differential equations are used to describe the dynamic behaviors of concentrations of biological components (*e.g.*, metabolites, mRNA, and peptides) and are solved by a set of differential equations with defined kinetic parameters of biological reactions or processes [51]. To explicitly solve such model, defined kinetic parameters are necessary, which are commonly estimated by fitting the experimental data with kinetic models. With the estimated parameters, the dynamic responses of objective biological components can be simulated in specific conditions. However, one of the limitations of the kinetic model is the difficulty in obtaining the kinetic parameters, especially the intracellular kinetic parameters. Ensemble modeling [52], a novel computational approach constructing the ensemble of all kinetic models with the same steady state, has been developed to analyze the kinetics allowable by thermodynamics and to further facilitate the strain design for metabolic engineering [53], [54], [55], [56], [57]. All approaches have been applied in *in vivo* metabolic engineering with tremendous success for rational design of the host cell [45], [48]. However, only a few pioneered studies aim at developing computational modeling approaches to predict the behaviors of *in vitro* synthetic systems, even with the fact that *in vitro* synthetic systems could be easier and more precisely described *via* kinetic models compared to *in vivo* systems [45], [46]. Recently, a non-linear kinetic model was used to describe the dynamic behavior of a SyPaB system, which was able to convert the glucose and xylose from corn stover to H_2_ and CO_2_, by estimating the kinetic parameters with the best fitting of experimental data [39]. The key enzymes with the largest impact of the final hydrogen yield and rate were identified by a global sensitivity analysis based on the kinetic model. By tuning enzyme loading based on the identified key enzymes, the volumetric hydrogen productivity was improved ~ 3-fold [39]. This improvement demonstrated the value of computational modeling approach to the SyPaB system. In addition to enhancing the performance of SyPaB systems, computational modeling of cell-free synthetic enzyme system was also able to help derive and test new modeling approach [45], [46]. A cutting-edge study attempted to derive a genome-scale cell-free kinetic modeling approach to simulate the biosynthetic capability of important industrial organisms (*e.g.*, *E. coli*) based on the advantages of kinetic modeling in cell-free synthetic enzyme systems [46]. In brief, the authors integrated complex allosteric regulations, which were encoded by simple effective rules and Hill-like transfer function, with traditional kinetic modeling. By modeling the kinetic profiles of several hypothetical cell-free metabolic networks, it was found that their integrated kinetic modeling approach could capture both the classic regulatory machinery (*i.e.*, product-induced feedback regulation) and the complex allosteric machinery (*i.e.*, non-competitive inhibition). Recently, a forward design method has been reported to establish an *in vitro* glycolysis biological process, which constituted of 10 enzymes [58]. The researchers combined online mass spectrometry and continuous system operation to apply standard system theory input functions and used the detailed dynamic system responses to parameterize a model of sufficient quality for forward design. This allows the facile optimization of a ten-enzyme cascade to produce an important intermediate in monosaccharide synthesis, dihydroxyacetone phosphate (DHAP) [58].

In summary, cell-free synthetic enzyme engineering is advantageous to *in vivo* metabolic engineering in speed, simplicity, and easiness of manipulation. However, there are still several drawbacks associated with cell-free synthetic enzyme engineering such as SyPaB. For example, in order to get the purified enzymes, researchers still need to spend numerous time and effort in plasmids construction, expression optimization and protein purification. Also, SyPaB was assembled in a complete artificial manner, which could lead to the instability of certain purified enzymes and coenzymes [8]. More importantly, the artificial environment could be dramatically different from the intracellular environment, which makes the results obtained from SyPaB optimization difficult to be transferred into *in vivo* metabolic engineering. The cell-free synthetic enzyme system itself, on the other hand, is arguably difficult in being scaled up for biomanufacturing [59], [60].

## 3 Cell-free Protein Synthesis (CFPS)-based Metabolic Engineering

A key difference between the cell-free synthetic enzyme engineering and the CFPS-based metabolic engineering is that the laborious *in vivo* protein expression and purification steps could be bypassed in the latter, which further speed up the DBT cycles. After decades of improving, current CFPS is well established, which could yield 200–2300 mg/mL protein in the batch mode reaction [61], [62], [63], [64], [65], [66], [67], [68], [69] and allow the CFPS-based metabolic engineering. Recently, Jewett et al. [20] reported this novel CFPS-based metabolic engineering framework for building biosynthetic pathways by directly synthesizing each enzyme of a biosynthetic pathway *in vitro* with the use of cell-free lysates and mixing multiple crude lysates to initiate the DBT cycle. A panel of cell-free lysates are selectively enriched and prepared in parallel, in each of which a target enzyme is overexpressed by using CFPS technology. Their cell-free lysates were next mixed in a combinatorial manner to construct a mevalonate biosynthetic pathway involved in isoprenoid synthesis [70]. Using this method, Jewett's group rapidly screened enzyme variants, optimized enzyme ratios, and explored cofactor landscapes for improving pathway performance. In the optimized system, mevalonate was synthesized at 17.6 g/L (119 mM) within 20 h compared to the initial titer of 1.6 g/L generated in 9 h. The fast prototyping and “debugging” of enzymatic pathways in this CFPS-based metabolic engineering framework offer unique advantages for metabolic engineering and synthetic biology applications because of the dramatically improved speed of DBT cycles. Encouraged by the successes of using CFPS-based metabolic engineering framework to produce mevalonate, this system was also applied to prototyping *n*-butanol biosynthesis [20]. It showed that *E. coli* lysates could support a highly active 17-step CoA-dependent *n*-butanol pathway derived from *Clostridia* metabolism involving CoA intermediates *in vitro* [20]. In this system, endogenous glycolytic enzymes convert glucose to acetyl-CoA for *n*-butanol synthesis, another *E. coli* enzyme (AtoB) converts acetyl-CoA to acetoacetyl-CoA, and heterologous enzymes (Hbd, Crt, Ter, AdhE) convert acetoacetyl-CoA to *n*-butanol. It was found that by adding both NAD and CoA with glucose to initiate n-butanol synthesis, the cell-free system could produce 1.2 g/L *n*-butanol. In order to improve pathway performance, the researchers replaced some of initial Ter and AdhE enzymes with a variety of homologs. In less than a day, they studied 4 Ter and 3 AdhE homologs by using CFPS-based metabolic engineering framework. Also they demonstrated the possibility of using linear DNA templates (*i.e.*, linear DNAs such as PCR products containing the whole expression cassette of the desired gene) instead of plasmids for pathway prototyping (*i.e.*, an early-stage method to study the constitution and function of a metabolic pathway), which would further expedite the process as the laborious cloning steps could be avoided. Finally, the *n*-butanol production was improved by 200% of the initial starting conditions (up to 1.5 g/L) by optimizing the performance of different enzymes' sets and adjusting the physicochemical environment.

Currently, no computational modeling approach has been reported to model the CFPS-based metabolic engineering framework [20], [71]. However, the CFPS-based metabolic engineering framework can be considered as the combination of two different procedures, *i.e.*, cell free protein synthesis and the SyPaB. In this case, it is possible to combine a CFPS model with the SyPaB models that are described in previous section to simulate and predict the performance of CFPS-based metabolic engineering. In spite of the unknown kinetic parameters of CFPS systems and the unclear composition of cell lysates [45], several studies have been implemented to develop various computational modeling approaches for both PURE system [72] and CFPS systems [72], [73], [74]. For example, one of the pioneered studies was recently implemented to derive a kinetic model to describe the gene expression dynamics in a commercial CFPS system producing green fluorescent protein (GFP) as the target [75]. By measuring the GFP expression and mRNA levels in the CFPS system, the authors estimated the unknown kinetic parameters in the model and predicted both DNA concentration and the experimental time as the key factors impacting the protein titer of CFPS [75]. In addition, computational models of CFPS systems can also elucidate the unknown impact of biological phenomena [45], [76]. In recent studies, it was found that increasing molecular crowding of CFPS system caused by crowding reagents or coacervation of encapsulated circuits, can improve the titer of protein production dramatically [76]. By modeling the transcription–translation reactions of CFPS system with the kinetic modeling approach, the author demonstrated that the improved protein production induced by coacervation was caused by the increased association constant of T7 polymerase as well as the kinetic transcription constant in the coacervated compartments [76]. Another hybrid kinetic model that combined a biological model with an agent-based model (or chemical kinetic model) has been developed to describe the *in vitro* protein synthesis and enabled the investigation of the polysome dynamics under the non-steady-state and non-continuum conditions [47]. We also want to point out that in addition to modeling the whole protein synthesis processes such as transcription and translation, many studies were also focusing on other bio-processes, *e.g.*, peptide chain elongation [77] and ribosome recycle [78], [79], which play pivotal roles in the entire protein synthesis.

When using kinetic models to simulate CFPS process, one of the major limitations is the ignorance of the transcriptional and translational regulations (*e.g.*, transcription factors) by using the kinetic parameters with constant values [80] to reflect time-dependent processes. In addition, the predictive capability of kinetic models is limited due to the unknown parameters [80]. Therefore, it is necessary to find an alternative algorithm with higher predictive capability to facilitate the design of CFPS systems. Machine learning, a central field of artificial intelligence, is an ideal choice for predictive analysis to devise complex systems with high non-linearity and multi-dimensionality [81], [82], [83]. Generally, machine learning can automatically learn the instinct correlations between the inputs and outputs of the systems, leading to a predictive model or algorithm with high prediction accuracy. For example, by training the machine-learning algorithm with paired inputs (*e.g.*, CFPS experimental designs and properties of target proteins) and outputs (*e.g.*, protein productions), the trained algorithm can predict the outputs from system inputs with high accuracy. Although CFPS is already simplified from the *in vivo* protein synthesis, it still has highly non-linear regulations and large-dimensional impact factors for the protein production [81]. Recently, a pioneering study has applied a machine learning algorithm (neural network) to the CFPS systems with paired data of different experimental designs and corresponding protein productions for learning CFPS systems and optimizing protein production [51]. The authors first set up a CFPS system to synthesize enhanced GFP (eGFP) by using commercial *E. coli* CFPS kits with fixed basic reaction system. Next, the authors chose 11 variable components in CFPS system and specified a vector of values for each component to build up a space of possible experiments. By using a robotic workstation for liquid handling, a larger number of CFPS experiments were implemented in a high-throughput manner [81]. Starting with randomly selected 49 experiments, the machine-learning algorithm started to learn the CFPS experiments and offered optimized designs of CFPS systems with improved eGFP production. With the optimized experimental design, the workstation implemented the next generation of experiments to generate new experimental data and to validate the predictions. By repeating this DBT cycles for eight times, the machine learning algorithm provided an optimized experimental design with ~ 3.5-fold improvement of eGFP production. Besides the improved protein production, the large-scale CFPS experiments and machine learning algorithm also uncovered kinetic biological insights to better understand the CFPS system [81]. This is the first time that machine learning algorithms have been integrated with CFPS systems without an arbitrary hypothesis, which demonstrates the capabilities and advantages of machine learning algorithms for better understanding the CFPS process.

## 4 Summary and Perspectives

Compared to traditional *in vivo* metabolic engineering, *in vitro* metabolic engineering has unique advantages in speeding up the DBT cycles. The key conceptual innovation of *in vitro* metabolic engineering is that the components in the DBT cycle can be purified enzymes or cell-free lysates rather than genetic constructs, thus avoiding engineering the complex cell metabolism and the tedious pathway construction. For the two *in vitro* metabolic engineering approaches discussed in this study, the major obstacles for cell-free enzymatic pathway engineering are the lack of stable building blocks as standardized parts and instability of costly coenzymes. By engineering thermo-stable enzymes and using them in *in vitro* metabolic engineering, the high productivity is likely to be maintained [8], [84]. CFPS-based metabolic engineering is arguably more advantageous because it could free the researchers from tedious protein purification and bypass the cofactor issues in a cytosol mimic environment. However, *in vitro* metabolic engineering approaches face the challenge of scaling up. Because of the high cost associated with the energy source (*e.g.* ATP) used in the cell-free system, the large-scale biomanufacturing is too expensive even when producing high-value products. Additionally, when using cell-free synthetic enzyme engineering, the stability of the enzymes could cause the reduced productivity during biosynthesis. Nevertheless, novel strategies from synthetic biology and protein engineering are being developed to address both challenges. For example, Caschera et al. have coupled polyphosphate and maltodextrin for bypassing substrate level phosphorylation based on expensive energy sources (phosphoenolpyruvic acid (PEP) and 3-PGA) [85]. Swartz et al. demonstrated that the costly NTP could be substituted with economic NMP and by shifting the energy source from expensive compounds to glucose. Thus, the cost–benefit of cell-free protein synthesis (g-product/$ reagent cost) is as much as 2.4 times higher than of reactions using costly PEP [67]. After decades' effort, cell-free protein synthesis could reach 2.3 mg/mL protein in the batch mode reaction which was comparable to *in vivo* expression levels [86]. Finally, although the scale-up of cell-free protein synthesis for *in vitro* metabolic engineering remains a challenge to be demonstrated, a milestone of the scale-up of CFPS has been achieved to expression complex high valued proteins in a 100 L reactor [59]. Refactoring the *in vitro* optimized pathway back into the host cells might be a future direction to address this scale-up problem. However, issues of lethality, toxicity of some metabolic intermediates and the compartmentalization of some pathways in the eukaryotic organisms should be aware during this process and might need additional DBT cycles to further improve the productivity. Meanwhile, to simulate and guide the design of *in vitro* metabolic engineering, data-driven algorithms (*e.g.*, machine learning and statistical learning) represent promising approaches, especially with the fast and high-throughput biological measurements of experimental data [83]. The data-driven algorithms can take advantage of the “Big Data” to uncover the biological insights behind the biological systems, and to derive the predictive models for predicting the outputs from corresponding inputs. Currently, one of the bottlenecks to develop the data-driven models is the limitation of high-quality and well-curated data [82]. Although several studies of *in vitro* metabolic engineering have been implemented and published, there is no database that curates these studies in a standardized manner, which obstructs the development of data-driven algorithms. The construction of such database requires both time and labors. However, it is still feasible to construct large-scale database including thousands of datasets in three to five years. With sufficient experimental data and appropriate data-driven algorithms, the internal complex interactions in the *in vitro* biological systems could be captured and explicitly elucidated in near future. It is worth noting that, biased data for training the data-driven algorithm will mislead the data-driven models. Therefore, using the equally distributed data to train the data-driven models is necessary to derive a data-driven algorithm with high prediction accuracy. To conclude, *in vitro* metabolic engineering, although still being on the infant stage, has great potentials in speeding up the DBT cycles of biomanufacturing and serves as an alternative approach to *in vivo* metabolic engineering.
